# Elevated depressive symptoms among newer and younger healthcare workers in Japan during the COVID‐19 pandemic

**DOI:** 10.1002/npr2.12217

**Published:** 2021-11-03

**Authors:** Narimasa Katsuta, Kanami Ito, Hiroshi Fukuda, Kuniaki Seyama, Satoshi Hori, Yuka Shida, Rie Nagura, Shuko Nojiri, Hiroyuki Sato

**Affiliations:** ^1^ Department of Psychiatry Juntendo University Faculty of Medicine Japan; ^2^ Department of Safety and Health Promotion Juntendo University Bunkyo‐ku Japan; ^3^ Department of General Medicine Juntendo University Graduate School of Medicine Bunkyo‐ku Japan; ^4^ Division of Respiratory Medicine Juntendo University Faculty of Medicine and Graduate School of Medicine Bunkyo‐ku Japan; ^5^ Medical Technology Innovation Center Juntendo University Bunkyo‐ku Japan

**Keywords:** clinical, COVID‐19, depression, epidemiology of mental disorders, healthcare workers, Japan, SARS‐COVID‐2

## Abstract

**Aim:**

Depression is a frequent outcome of long‐term stress, but no studies have examined depression rates among Japanese healthcare workers fighting the COVID‐19 pandemic. Therefore, we conducted a web‐based interview of hospital employees to assess depression prevalence and factors.

**Methods:**

This observational cohort study was conducted from July to August, 2020, as part of a mandatory health checkup of Juntendo University Hospital employees (Tokyo, Japan). A total of 4239 participants completed a web‐based questionnaire on medical history and current health status. The Center for Epidemiologic Studies Depression Scale (CES‐D) was used for self‐assessment, with a score of ≥16 considered to indicate depression.

**Results:**

Among all employees, the proportion of depression was 31.3% in 2020, the highest measured in the last 10 years and substantially greater than the pre‐pandemic value in 2019 (27.5%). The proportion of depression for 2020 was significantly higher in new recruits than in employees with more than 2 years of experience (47.0% vs 29.9%, respectively, *P* < .0001) and in new recruits in 2019 (26.4%, *P* < .0001). When subdivided by occupation, nurses demonstrated the highest depression rate (43.2%), followed by paramedics (35.1%) and clerks (31.6%), whereas residents (22.9%), doctors (20.4%), teaching staff (18.0%), and part‐time staff (15.3%) reported lower depression rates. The positive CES‐D score significantly correlated with age (*P* < .0001).

**Conclusions:**

Younger and newer employees demonstrated the highest rates of depression independent of occupation. Therefore, mental healthcare programs focusing on these vulnerable groups need to be established.

## Abstract 2

Younger and newer employees demonstrated the greatest rates of depression independent of occupation. Mental healthcare programs focusing on these vulnerable groups are required.

## Graphical abstract

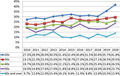

## 1 INTRODUCTION

In winter 2019, a new viral pneumonia was detected in Wuhan, Hubei Province, China, and the causative pathogen identified as severe acute respiratory syndrome coronavirus 2 (SARS‐CoV‐2). Within several months, the associated disease, COVID‐19, had spread throughout the globe. By the end of October, 2020, the total number of infected cases exceeded 100 000 in Japan, with more than 1700 deaths. The mental health of healthcare workers involved in COVID‐19 treatment is a critical factor for sustaining the global fight against this disease.

Several reports examining healthcare workers during SARS, MERS, and COVID‐19 pandemics have found substantial impacts on physical and mental health, including increased susceptibility to insomnia.^1^, ^2^, ^3^ Healthcare workers around the world have experienced significant psychological stress and will require care to continue to protect people from COVID‐19.^4^ The impact of the COVID‐19 pandemic has recently become clear in terms of impairing psychological health and exacerbating the risk of suicide because many problems of COVID‐19 are protracted. In Japan, from July 2020 to October 2020, the monthly suicide rate increased by 16%, which further increased among women, children, and adolescents.^5^ The mental health of the entire population was adversely affected; however, the mental health problems of healthcare workers were even more serious. Psychological distress was reported to significantly increase in healthcare workers, in addition to fear and anxiety regarding COVID‐19.^6^ According to a July 2020 report, the prevalence of depressive symptoms in Japan was two to nine times higher than that before the COVID‐19 pandemic.^7^ Depression is a frequent outcome of long‐term stress, but no studies have examined depression rates among Japanese healthcare workers fighting the COVID‐19 pandemic. Therefore, we conducted a web‐based interview of hospital employees to assess depression prevalence and factors associated with greater depression risk.

## 2 METHODS

This observational cohort study was conducted from July to August, 2020, as part of a mandatory health checkup of Juntendo University Hospital employees (Tokyo, Japan). All 4,290 employees were enrolled. The healthcare staff who were assessed included doctors, residents, nurses, paramedics, support staff, clerk, teaching staff, researchers, and part‐time staff. Two study subjects who opted were thus excluded. A total of 4239 participants completed a web‐based questionnaire on medical history and current health status, and the valid response rate obtained was 98.8%. The Center for Epidemiologic Studies Depression Scale (CES‐D) was used for self‐assessment, with a score of ≥16 considered to indicate depression.

The study protocol was approved by the Ethics Committee of the Juntendo University Faculty of Medicine (approval no. 22004). Informed consent was obtained from all participants.

Statistical analyses were performed using SPSS ver. 22 (IBM Corp.). The chi‐square test was used to assess differences in the frequencies of patient characteristics (eg, sex). Clinical variables were compared using the two‐tailed Mann‐Whitney U test in cases with two groups or the Kruskal‐Wallis test in cases with three or more groups. A two‐tailed *P* < .05 was considered significant for all tests.

The correlation between the participants’ variables was analyzed by univariate analysis. The analysis was performed using only the variables associated with the CES‐D scores. Logarithmic conversion was performed for age. The models included multiple co‐variates such as gender, age, observation year, and healthcare occupation. Observation year and healthcare occupation were tested in separate models including interaction terms of all the above‐mentioned co‐variates. The type used in the models was “nurse vs. non‐nurse.” Logistic regression analysis was performed subsequently using multiple categories as outcome variables (ie, CES‐D‐positive findings) to identify correlations between different clinical factors and depression.

## 3 RESULTS

**FIGURE 1:**
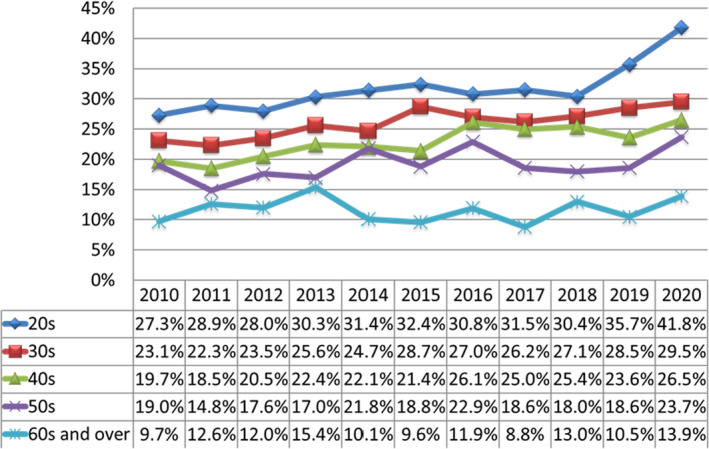
Changes in depression prevalence among employees of Juntendo University Hospital and Juntendo University Graduate School of Medicine according to age group over the past decade. Depression was evaluated by the Center for Epidemiologic Studies Depression Scale (CES‐D). A score of ≥16 was considered positive for depression. Depression has remained highest in the youngest age group (20s, blue), with greatest prevalence during the COVID‐19 pandemic year of 2020

The prevalence of depression among all employees was 31.3% in 2020, the highest measured in the last 10 years and substantially greater than the pre‐pandemic value in 2019 of 27.5%. The proportion of depression for 2020 was significantly higher in new recruits than in employees with more than 2 years of experience (47.0% vs 29.9%, respectively, *P* < .0001) and in new recruits in 2019 (26.4%, *P* < .0001). Depression was the highest in the 20s age group (Figure 1). When the participants were subdivided by occupation (nurses, paramedics, doctors, residents, clerks, researchers, support staff, teaching staff, and part‐time staff), nurses had the highest depression rate (43.2%), followed by paramedics (35.1%), clerks (31.6%), support staff (28.9%), and researchers (27.6%), whereas residents (22.9%), doctors (20.4%), teaching staff (18.0%), and part‐time staff (15.3%) reported lower depression rates (Table 1).

**TABLE 1:** Comparison of 2020 and 2019 CES‐D results for Juntendo University Hospital employees (Tokyo, Japan)

	Outcome of CES‐D		
	2020	2019	
	N = 4238	N = 4240	*P*‐value
Sex, M/F	1555/2683	1633/2607	.083
Age (Y)	37.6 ± 12.1	37.8 ± 12.1	.413
Number and the positive rate of CES‐D by occupation			
Doctor	606 (20.4%)	611 (18.0%)	.309
Resident	96 (22.9%)	98 (19.4%)	.600
Nurse	1314 (43.2%)	1295 (40.0%)	.095
Paramedics	519 (35.1%)	501 (30.5%)	.126
Support staff	76 (28.9%)	100 (30.0%)	1.000
Clerk	437 (31.6%)	397 (28.7%)	.406
Teaching staff	256 (18.0%)	241 (14.9%)	.398
Researcher	653 (27.6%)	648 (20.5%)	.**003**
Part‐time	281 (15.3%)	349 (15.2%)	1.000
Average score by occupation			
Doctor	9.0 (4.0 ‐ 13.0)	6.0 (2.0 ‐ 12.0)	**<.001**
Resident	11.0 (5.0 ‐ 15.0)	6.5 (2.0 ‐ 12.25)	.**013**
Nurse	14.0 (9.0 ‐ 21.0)	13.0 (6.0 ‐ 20.0)	**<.001**
Paramedics	12.0 (7.0 ‐ 19.0)	10.0 (5.0 ‐ 17.0)	.**002**
Support staff	12.0 (8.0 ‐ 17.0)	10.0 (5.25 ‐ 7.75)	.238
Clerk	11.0 (6.0 ‐ 18.0)	9.0 (4.0 ‐ 16.5)	.**01**
Teaching staff	7.0 (4.0 ‐ 13.0)	5.0 (2.0 ‐ 11.0)	**<.001**
Researcher	10.0 (6.0 ‐ 17.0)	8.0 (3.0 ‐ 13.75)	**<.001**
Part‐time	7.0 (3.0 ‐ 12.0)	5.0 (2.0 ‐ 11.0)	.**017**

Note

*P* values with statistical significance are in bold.

Abbreviation: CES‐D, The center for epidemiologic studies depression scale.

Center for Epidemiologic Studies Depression Scale significantly and positively correlated with age (*P* < .0001) and sex (*P* < .0001). In addition, occupation was associated with score changes between 2019 and 2020 (*P* < .0001). The results from binary logistic regression analysis for the score changes between 2019 and 2020 showed that being female [odds ratio (OR): 1.22; 95% confidence interval (CI): 1.09‐1.38], younger (OR: 2.14; 95% CI: 1.77‐2.59), a nurse (OR: 2.60; 95% CI: 2.03‐3.34), paramedic (OR: 2.16; 95% CI: 1.66‐2.80), support staff (OR: 2.15; 95% CI: 1.45‐3.19), clerk (OR: 2.05; 95% CI: 1.57‐2.68), or researcher (OR: 1.41; 95% CI: 1.09‐1.83), and the results being in 2020 (OR: 1.19; 95% CI: 1.08‐1.31) were significantly associated with positive CES‐D findings. In addition, we evaluated the association between year and occupation (nurse vs. non‐nurse) using the following formula:

$$Y=\beta_{0}\times occupation\left(\mathrm{nurse}\right)+\beta_{1}\times year+\beta_{2}\times age+\beta_{3}\times \;\text{sex}\;+\beta_{4}\times occupation\left(\mathrm{nurse}\right)\times year$$

No significant interaction was observed between year and occupation (*p* = 0.939).

## 4 DISCUSSION

No study has yet investigated the effects of the COVID‐19 pandemic on Japanese healthcare employees according to years of experience. However, a recent systematic review on depression in the general population reported a greater depressive symptom rate among the younger age group (≤40 years) compared to the older group (≥40 years).^8^ It is possible that younger age per se may account at least in part for the higher depression rate among new employees.

Juntendo University Hospital is an advanced treatment hospital and has accepted critical patients with COVID‐19 because it can provide advanced medical care. The turmoil in Tokyo at that time was tremendous, and caring for the severely ill was a source of great psychological stress for healthcare workers. The higher depression rate among nurses and paramedics may result from the greater risk of infection when working in close proximity to COVID‐19 patients, especially in cases where patients are unmasked or personal protective equipment (PPE) is inadequate. This stress may be further compounded by a relative lack of experience treating contagious patients. The higher depression rate among research students may result from economic concerns as these students are poorly paid, and economically vulnerable individuals generally demonstrate poorer mental health.^9^ For all employees, better training, access to PPE, adequate rest, and both practical and psychological support should help improve psychological outcomes.^10^ Based on the current results, these interventions appear particularly important for students, paramedics, and nursing staff.

During respiratory illness epidemics, additional work‐related stressors increase. Nurses are especially vulnerable to the risk of infection because they often work at frontlines to provide care to patients; therefore, they have high level of occupational stress. However, in our analysis, no statistical significance was observed between occupation (nurse vs. non‐nurse) and the COVID‐19 pandemic year. Most of the occupations in this study were healthcare‐related, thus constant exposure to the risk of infection and fear and anxiety resulting from it would naturally be higher in these occupations than in non‐medical ones.

The limitations of this study include the observational study design and reliance on a single depression scale (CES‐D). For example, we did not examine in greater detail the factors related to occupational stress as well as other significant psychological stressors linked to the work environment including dealing with death and dying, inadequate emotional preparation, social stigma, isolation, and uncertainty concerning the treatment. Follow‐up studies including the results from other centers and more extensive test batteries are required to improve the applicability of these findings to the broader population of healthcare workers involved in treating COVID‐19 patients.

In conclusion, nurses and paramedics demonstrated the highest rates of depression among employees of Juntendo University Hospital and Juntendo University Graduate School of Medicine during 2020, possible due to the risks involved in directly treating COVID‐19 patients. In addition, younger and newer employees demonstrated the greatest rates of depression independent of occupation. Mental healthcare programs focusing on these vulnerable groups are required.

## 5 APPROVAL OF THE RESEARCH PROTOCOL BY AN INSTITUTIONAL REVIEWER BOARD

The study protocol was approved by the Ethics Committee of the Juntendo University Faculty of Medicine (Approval no. 22004).

## CONFLICT OF INTEREST

The authors declare no conflicts of interest.

## AUTHOR CONTRIBUTION

All authors contributed to the conceptualization, design, and writing of this manuscript.

## INFORMED CONSENT

Informed consent was obtained from all participants.

## Data Availability

Research data are not shared. The raw data belonged to the present study cannot be made publicly available because the disclosure of personal data was not included in the research protocol of the present study. The data are not publicly available due to privacy and ethical restrictions.
